# Oral and dermal exposure to natural radionuclides and heavy metals in water and sediments of Nile River, Qena, southern Egypt

**DOI:** 10.1038/s41598-023-49389-3

**Published:** 2023-12-13

**Authors:** Khaled Salahel Din, Faten Mahmoud

**Affiliations:** 1 Physics Department, Faculty of Science, South Valley University, Qena, 83523 Egypt; 2 Health Ministry Labs., Qena Branch, Qena, Egypt

**Keywords:** Natural hazards, Physics, Environmental sciences, Environmental impact

## Abstract

This study assessed the levels of natural radionuclides (^226^Ra, ^232^Th, and ^40^K) and heavy metals (Hg, Fe, Cr, As, Zn, Cu, Cd, and Pb) in surface water and sediment samples from the Nile River in Qena Governorate, southern Egypt, using a gamma-ray spectrometer, 3ʺ NaI (Tl) scintillation detector coupled with 1024 multi-channel analyzer, and an atomic absorption spectrometer. In surface water and sediments, the average activity concentrations of natural radionuclides were ^40^K (4.73 Bq L^−1^; 395.76 Bq kg^−1^) > ^226^Ra (0.41 Bq L^−1^; 18.14 Bq kg^−1^) > ^232^Th (0.30 Bq L^−1^; 17.98 Bq kg^−1^). The average heavy metal concentrations in surface water in µg L^−1^ were Fe (121.0) > Zn (33.80) > Cr (28.0) > Cu (8.62) > Pb (8.35) > As (1.19) > Hg (0.81) > Cd (0.12). In Nile sediments the concentrations in mg kg^−1^ were Fe (1670.0) > Zn (207.0) > Cr (29.40) > Cu (16.20) > Pb (4.32) > Hg (0.41) > Cd (0.31) > As (0.14). The heavy metal evaluation index (HMEI) calculations for water samples revealed that 31% of the samples were suitable for domestic use, while 69% were not. The geo-accumulation index, enrichment factor, and ecological risk factor for sediments were estimated, showing extreme enrichment for Hg and Zn with high ecological risk for Hg. Health risks for adults were evaluated due to oral and dermal exposure to Nile surface water and sediments from the study area, indicating minimal radiological risks and potential carcinogenic and non-carcinogenic risks from the metals.

## Introduction

Natural radionuclides, such as ^40^K and members of the ^238^U and ^232^Th series, are found throughout our environment in varying levels across rocks, soils, water, air, and plants ^1^. Radionuclides and contaminants can be released into aquatic environments, where they disperse and transport through turbulent processes in the water phase. In these environments, radionuclide content depends on the mineral characteristics of the catchment area and aquatic environment properties, such as pH, organic matter content, and redox potential ^2^. Radionuclides are typically present in aquatic environments at extremely low concentrations, ranging from 10^−15^ to 10^−20^ molars, but in some cases, levels of natural radioactivity can reach up to 10^3^ Bq kg^−1^ ^3^.

The contamination of water bodies by heavy metals (HMs) has become a global concern due to the progress of industrial development and population growth. HMs possess high toxicity, are non-biodegradable, and exhibit bioaccumulation behavior, posing harmful effects on human and aquatic life. Consequently, elevated concentrations of HMs in water bodies render the water unsuitable for various purposes, including drinking and irrigation. Although HMs can naturally occur in surface water resources, anthropogenic activities such as industrial and domestic effluents, mining operations, and agricultural practices serve as the primary sources of HMs. Consequently, the concentrations of HMs in unaffected water bodies are typically minimal and predominantly originate from the weathering of rocks and soils ^4–6^.

Rivers are highly vulnerable to both anthropogenic and natural contamination. While, they are naturally influenced by factors like precipitation, weather conditions, and sediment transport, human activities can exacerbate the negative ecological impacts on these water sources. Collecting reliable data on river water quality, assessing its spatial and temporal variations, identifying contamination sources, evaluating water quality status, and monitoring water pollution in rivers are crucial for effective water management ^5^.

Sediments play a critical ecological role in rivers. However, they also act as a reservoir for radionuclides and HMs. When these pollutants enter rivers, a small portion remains dissolved in the water, while a large portion is deposited in the sediment. However, sediment can act as a secondary source of pollution by releasing pollutants through chemical, physical, and biological processes. Moreover, sediments provide important historical records of changes caused by human activities and natural watershed processes due to their long residence time. Therefore, sediment analysis is a valuable tool for determining the level of pollution in rivers ^3,4^.

The Nile River, the second-longest river globally and the longest in Africa, has a basin area of 2.9 million km^2^. It passes through 10 African countries, with a mainstream length of about 6700 km, and its total length, including tributaries, reaches up to 37,205 km ^7–9^. In Egypt, the Nile extends for 950 km from Aswan to north of Cairo, serving as the primary water source for various uses, including drinking, household supply, agriculture, industry, navigation, and fishing.

Nile water quality is a major concern in Egypt due to the increasing pollution sources that discharge waste into the river. The Nile receives wastewater from over 90 drains spread from Aswan in the south to its outlet in the Mediterranean Sea in the north, including agricultural, industrial drains, and domestic sewage effluents ^9,10^. Soil erosion and leaching processes also contribute to the presence of pollutants in the Nile water. Furthermore, the impact of the Ethiopian dam construction on the Nile water level and the resulting effect on pollution levels pose a challenge for the country.

The assessment of the Nile River's suitability for drinking and aquatic life is crucial. The growth of industrial and agricultural activities in Qena Governorate, located in southern Egypt, results in the release of significant amounts of untreated wastewater into the Nile, raising concerns about possible high levels of pollutants. This study aims to evaluate the pollution level and potential health risks associated with exposure to natural radionuclides (^226^Ra, ^232^Th, and ^40^K) and HMs (Hg, Fe, Cr, As, Zn, Cu, Cd, and Pb) in the water and sediments of the Nile River along Qena Governorate.

## Material and methods

### Study area and sampling

**Figure 1:**
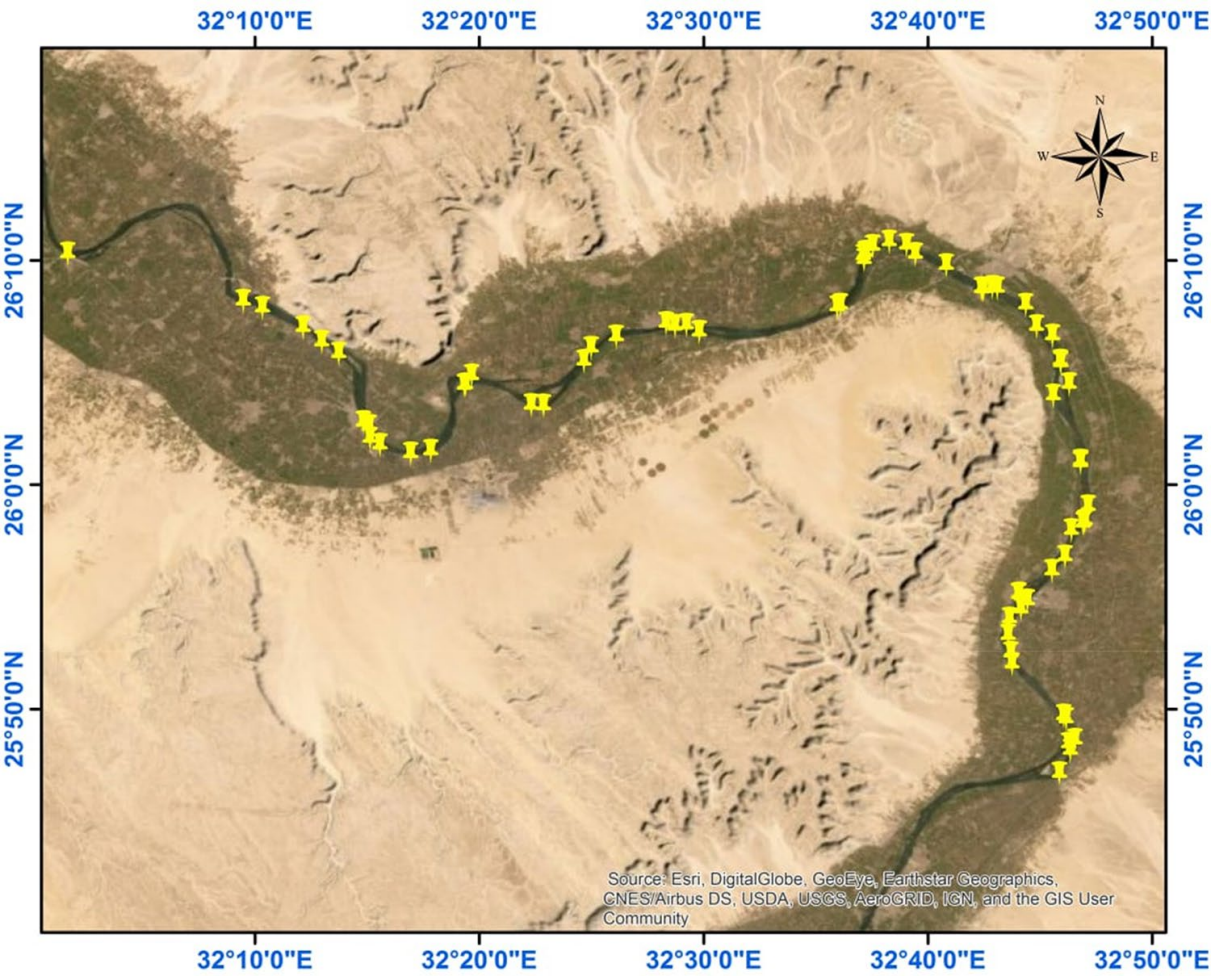
Sampling sites along Nile River from Qena governorate, south Egypt (ArcGIS software 10.8.1; ArcGIS Online).

The Qena governorate, an Egyptian province situated approximately 600 km south of Cairo, spans a land area of around 10,798 km^2^ and has a population of about 3.3 million people. It lies between latitudes 26° 15ʹ N and 26° 8ʹ N and longitudes 32° 50ʹ E and 32° 42ʹ E ^11^. 70 water samples and 20 sediment samples were collected randomly during May–June 2019 from various points in the Nile River, stretching from Qus city in the south to Abu Teshit city in the north (Fig. 1). Water samples were collected 1 m below the surface using a closed-cap bottle attached to a 2 m wire, opening the cap at the desired depth. The samples were filtered with filter paper to remove suspended particles and acidified to a pH of ≤ 2 using HNO3 drops. The samples were then filled in sealed 1.4-L Marinelli containers ^11,12^.

Sediment samples weighing 1 kg each were obtained using an Ekman grab sediment sampler and stored in polyethylene bags. These samples were oven-dried at 105 °C for 24 h to eliminate moisture and achieve a constant weight before being crushed, homogenized, and sieved through a 2 mm mesh. The dried samples were placed in airtight 500 mL Marinelli containers. Both water and sediment samples were stored for one month to establish equilibrium between radon products and their parent elements before gamma spectrometer measurements^13^.

### Radioactivity measurements

The specific activity of ^226^Ra, ^232^Th, and ^40^K was determined using a gamma-ray spectrometer composed of a three-inch NaI (Tl) scintillation detector connected to a 1024 multi-channel analyzer. The detector was housed within a 100 mm thick cylindrical lead shield with a fixed bottom and movable cover, featuring an inner 0.3 mm thick copper cylinder. Energy calibration employed standard ^60^Co and ^137^Cs point sources, while efficiency calibration utilized a multi-radionuclide standard source, covering an energy range of 59.54–1836 keV. Detailed calibration procedures can be found in previous work ^14^. Each sample was measured for 86,400 s to minimize statistical uncertainty, and the spectra were analyzed using Maestro computer software. ^226^Ra was estimated from γ-lines of ^214^ Bi (609 keV) and ^214^Pb (352 keV). ^232^Th was from γ-lines of ^228^Ac (911 keV). ^40^K was from its single γ-line of 1460 keV. Specific activity calculations used Eq. (1) ^15^.

1 $${\text{A}}=\frac{{C}_{S}-{C}_{B}}{{I}_{\upgamma }\cdot {\varepsilon }_{\upgamma }\cdot t\cdot m \left(or\right)v}$$

where A is specific activity (Bq kg^−1^) or (Bq L^−1^), C_S_ and C_B_ are the peak counts at energy E for the sample and background, respectively, I_ɤ_ and *ε*_*ɤ*_ are the intensity of gamma rays and detection efficiency at energy E, respectively, t is live time in second and m is the sample mass in kilogram, v is sample volume in liter. The minimum detectable activity (MDA) was calculated according to Curie definition using Eq. (2)^16^.

2 $${\text{MDA}}=\frac{2.71+4.65\sqrt{B}}{{I}_{\upgamma }\cdot {\varepsilon }_{\upgamma }\cdot t\cdot m}$$

where B is the background count in radionuclide spectrum. The uncertainty of specific activity (U_A_) was calculated using Eq. (3)^17^.

3 $$ U_{A}  = \sqrt {\left( {\frac{{UN_{S} }}{{N_{S} }}} \right)^{2}  + \left( {\frac{{UN_{B} }}{{N_{B} }}} \right)^{2}  + \left( {\frac{{U\varepsilon _{\gamma } }}{{\varepsilon _{\gamma } }}} \right)^{2}  + \left( {\frac{{U_{m} }}{m}} \right)^{2} }  $$

where UN_s_ is sample count uncertainty, UN_B_ is background count uncertainty, U${\varepsilon }_{\gamma }$ is detector efficiency uncertainty, and U_m_ sample mass uncertainty.

### Heavy metals measurements

For water samples, 200 ml of sample was digested with 10 ml of concentrated nitric acid (HNO_3_) and evaporated on a hot plate until near dryness, approximately 5 ml. The samples were cooled to room temperature, filtered through a 0.45 µm filter paper, and deionized water was added to the filtrate until it reached a volume of 25 ml ^18^.

For sediment samples, 2 g of dried sample was placed in a Teflon container and mixed with 8 ml of 65% HNO_3_ using an automatic mixer (Vortex Mixer MVOR-03) for 10 min. The container was then placed in a microwave digestion oven (Speed Wave B-ERGHOF). After digestion, the digested sample was filtered through filter paper, and deionized water was added to the filtrate to reach a volume of 25 ml ^19^.

The contents of Hg, Fe, Cr, As, Cu, Zn, Cd, and Pb in the prepared water or sediment samples were analyzed using an AAS (atomic absorption spectrometer, Perkin Elmar AAnalyst 700) at specific wavelengths. Calibration was achieved by preparing standard metal solutions, dissolving the standard stock solution of each metal with 2% nitric acid and diluting with deionized water to optimal concentrations in 100 ml volumetric flasks. Calibration curves were obtained for each element by plotting the concentrations of the standards against their absorbance. Blank samples were prepared using the same reagents but without the metal stock solutions ^19,20^. The accuracy of the AAS was evaluated by the analysis of certified reference material (PACS-3, marine sediments). A recovery rate of 89–96% was acquired and thought satisfactory for analysis (Table S1).

### Assessment of metals pollution

#### Contamination indices of water samples

##### Water quality index (WQI)

To evaluate the Nile water's suitability for drinking, the Water Quality Index (WQI) was determined using Eq. (4)^21^.

4 $${\text{WQI}}=\frac{\sum_{{\text{i}}=1}^{{\text{n}}}{{\text{W}}}_{{\text{i}}}{{\text{Q}}}_{{\text{i}}}}{\sum_{{\text{i}}=1}^{{\text{n}}}{{\text{W}}}_{{\text{i}}}}$$

here, W_i_ represents the weighting factor, and Q_i_ signifies the individual quality rating of the i-th heavy metal. These values are determined by Eqs. (5)–(7)^21,22^.

5 $${{\text{W}}}_{{\text{i}}}=\frac{K}{{C}_{standard}}$$

where K is the proportionality constant defined as:

6 $$ {\text{K}} = \frac{1}{{\sum {\left( {\frac{1}{{C_{{standard}} }}} \right)} }} $$

C_standard_ represents the standard value of the ith heavy metal concentration for drinking water, obtained from the Egyptian Drinking Water Quality Standards (EWQS) and the World Health Organization (WHO) ^23,24^.

7 $${{\text{Q}}}_{{\text{i}}}=\frac{({C}_{actual}-{C}_{ideal})\times 100}{({C}_{standard}-{C}_{ideal})}$$

C_actual_ is the measured value of i-th heavy metal concentration in the sample, while C_ideal_ is the ideal value of the i-th heavy metal concentration in pure water, assumed to be zero for all studied heavy metals. The index values are rated as follows: 0–25 (excellent), > 25–50 (good), > 50–75 (poor), > 75–100 (very poor), and > 100 (unsuitable) ^22^.

##### Heavy metal evaluation index (HMEI)

Water contamination levels were assessed by calculating the Heavy Metal Evaluation Index using Eq. (8)^25^.

8 $${\text{HMEI}}=\sum_{i=1}^{n}\frac{{C}_{actual}}{{C}_{standard}}$$

C_actual_ and C_standard_ are defined as above. The HMEI limit value is set to one; water with an HMEI value < 1 is considered fit for domestic use, while water with a value > 1 is unfit ^22,25^.

#### Contamination indices of sediment samples

##### Geo-accumulation index (I_geo_)

To evaluate the level of metal contamination in Nile sediments, the Geo-accumulation Index (I_geo_) was determined using Eq. (9)^26^.

9 $${{\text{I}}}_{{\text{geo}}}={{\text{Log}}}_{2}\frac{{{\text{C}}}_{{\text{i}}}}{{{\text{KB}}}_{{\text{i}}}}$$

C_i_ is the measured concentration of i-th heavy metal in the sample, B_i_ is the geochemical background value of i-th metal, and k = 1*.*5 is the background correction factor accounting for possible differences in background values. Metal concentrations in the Upper Continental Crust (UCC) from Wedepohl were used as background values ^27^.

##### Enrichment factor (EF)

To identify potential natural or anthropogenic sources of HMs in sediments, the Enrichment Factor is calculated using Eq. (10)^28^.

10 $$EF=\frac{\left(\frac{M}{R}\right)sample}{\left(\frac{M}{R}\right)UCC}$$

where (M/R)_sample_ is the concentration ratio measured in the sample for the metal of interest M and the reference metal R (Fe), and (M/R)_UCC_ is the metal concentration ratio in the upper continental crust (UCC). Fe was utilized as a reference metal in EF calculation because it is a major abundant element in the earth's crust and has no anthropogenic contribution ^28^.

##### Ecological risk factor (E_ri_)

The potential environmental risks posed by certain pollutants in sediments can be expressed by calculating the Environmental Risk Factor (E_ri_) using the method proposed by Hackanson, as shown in Eq. (11)^29^.

11 $${{\text{E}}}_{{\text{ri}}}={T}_{ri }\times \frac{{C}_{i}}{{C}_{UCC}}$$

T_ri_ is the toxic response factor of the ith heavy metal (i.e. Hg = 40, Cd = 30, As = 10, Pb = Cu = 5, Cr = 2, Zn = 1, Cr = 2) ^30^, C_i_ is the concentration of the i-th heavy metal in sample, and C_UCC_—is the i-th metal concentration in the upper continental crust ^27^. Iron (Fe) was excluded from the calculation due to its low potential environmental risks ^31^. Threshold values of the contamination indices and their corresponding contamination degrees are summarized in Supplementary Table S2.

### Human health risk assessment

The potential human risks for adults due to direct ingestion and dermal exposure pathways of natural radionuclides and HMs were assessed. It is worth noting that only the oral exposure risk assessment was considered for radionuclides because the skin acts as a barrier against radionuclide absorption, making dermal exposure contributions minimal ^32^.

### Radiological risk assessment

The Annual Effective Dose (AED) resulting from oral exposure of natural radionuclides in Nile River water and sediments was estimated for adults, considering ingestion rates of 2 l day^−1^ and 100 mg day^−1^ for water and sediment, respectively ^24,33^ and using the following equation^34^.

12 $$ AED = A \times I_{R} \times C_{F} \times E_{F} $$

AED refers to the annual effective dose in mSv year^−1^, A represents the radionuclide activity concentration in Bq L^−1^ or Bq kg^−1^, I_R_ denotes the daily ingestion rates for adults in L day^−1^ or kg day^−1^, C_F_ represents the dose conversion factors 2.8 × 10^−7^, 2.3 × 10^−7^, and 6.2 × 10^−9^ Sv Bq^−1^ for ^226^Ra, ^232^Th, and ^40^ K, respectively ^35^, E_F_ is exposure frequency, which is 365 day year^−1^^33^.

Carcinogenic risk can be estimated by multiplying the daily intake of each radionuclide (DI = A × I_R_) with its cancer slope factor and the average exposure time (70 years) according to Eq. (13)^36^.

13 $$ {\text{ELCR}} = {\text{DI}} \times {\text{CSF}} \times {\text{AT}} $$

ELCR refers to the probability of devolving cancer in individuals over their lifetime (unitless quantity), DI denotes the daily intake of radionuclides (Bq day^−1^), CSF represents the cancer slope factor indicating the morbidity risk (pCi risk^−1^) provided by United State Environmental Protection Agency (US-EPA) ^36^, and AT stands for the average exposure time in days (70 years × 365 days). Since the slope factor was given in pCi risk^−1^ and the intake was in Bq, the activity was converted from Bq to pCi using the conversion factor of 1 Bq = 27 pCi.

#### Heavy metal risk assessment

To evaluate the potential adverse health effects on adults due to HMs exposure from Nile River water and sediment through oral and dermal absorption pathways, three factors must be estimated: chronic daily intake (CDI), hazard quotient (HQ), and hazard index (HI). These factors are described by the US-EPA using Eqs. (14)–(17)^33,37,38^.

14 $${{\text{CDI}}}_{{\text{or}}}=\frac{{C}_{i \times IR\times EF\times ABS\times ED}}{BW\times AT}$$

15 $${{\text{CDI}}}_{{\text{de}}}=\frac{{C}_{i \times SA\times {K}_{p}\times ABS\times EF\times ED\times ET\times CF}}{BW\times AT}$$

16 $${{\text{HQ}}}_{{\text{or}}/{\text{de}}}=\frac{{CDI}_{in/d}}{{RfD}_{in/d}}$$

17 $$ {\text{HI}}_{{{\text{or}}/{\text{de}}}}  = \sum\limits_{{i = 1}}^{n} {HQ_{{in/d}} }  $$

where: CDI_or/de_ refers the chronic daily intake of the HMs due to the oral/dermal absorption pathways (mg kg^−1^ day^−1^). HQ_or/de_ represents the hazard quotient, indicating the potential toxicity posed by the HMs via oral/dermal absorption pathways (unitless quantity). RfD_or/de_ denotes the reference dose, defined as the maximum acceptable dose of a HMs without human health risks due to the oral/dermal absorption pathways (mg kg^−1^ day^−1^). HI_or/de_ stands for the hazard index, showing the total potential non-carcinogenic health risks due to oral/dermal absorption pathways exposures to all tested HMs in water/sediment (unitless quantity).

The US-EPA set a threshold value of 1.0 for HI. If HI exceeds 1.0 for the exposure scenario, the exposed person may be subject to non-carcinogenic health risks ^37^. All parameters used in the risk assessment calculations, along with their definition, reference values, and units are provided in Supplementary Table S3.

Carcinogenic health risks were estimated using the incremental lifetime cancer risk (ILCR), defined as the increased likelihood of an exposed person developing cancer over their lifetime due to exposure to a given daily amount of a carcinogenic element (in this study: Cr, As, Cd, and Pb). ILCR was calculated for adults according to Eq. (18)^33^.

18 $$ {\text{ILCR}} = {\text{CDI}}_{{\text{or/de}}} \times {\text{CSF}} $$

where CDI_or/de_ is as defined earlier, and CSF (mg kg^−1^ day^−1^)^−1^ represents the risk generated by a lifetime average amount of one mg kg^−1^ day^−1^ of a specific carcinogen heavy metal (Table S2). ILCR values above 1.0 E − 04 are considered unacceptable and signify a potential cancer risk to humans ^36^.

## Results and discussion

### Natural radionuclides levels in Nile River

**Figure 2:**
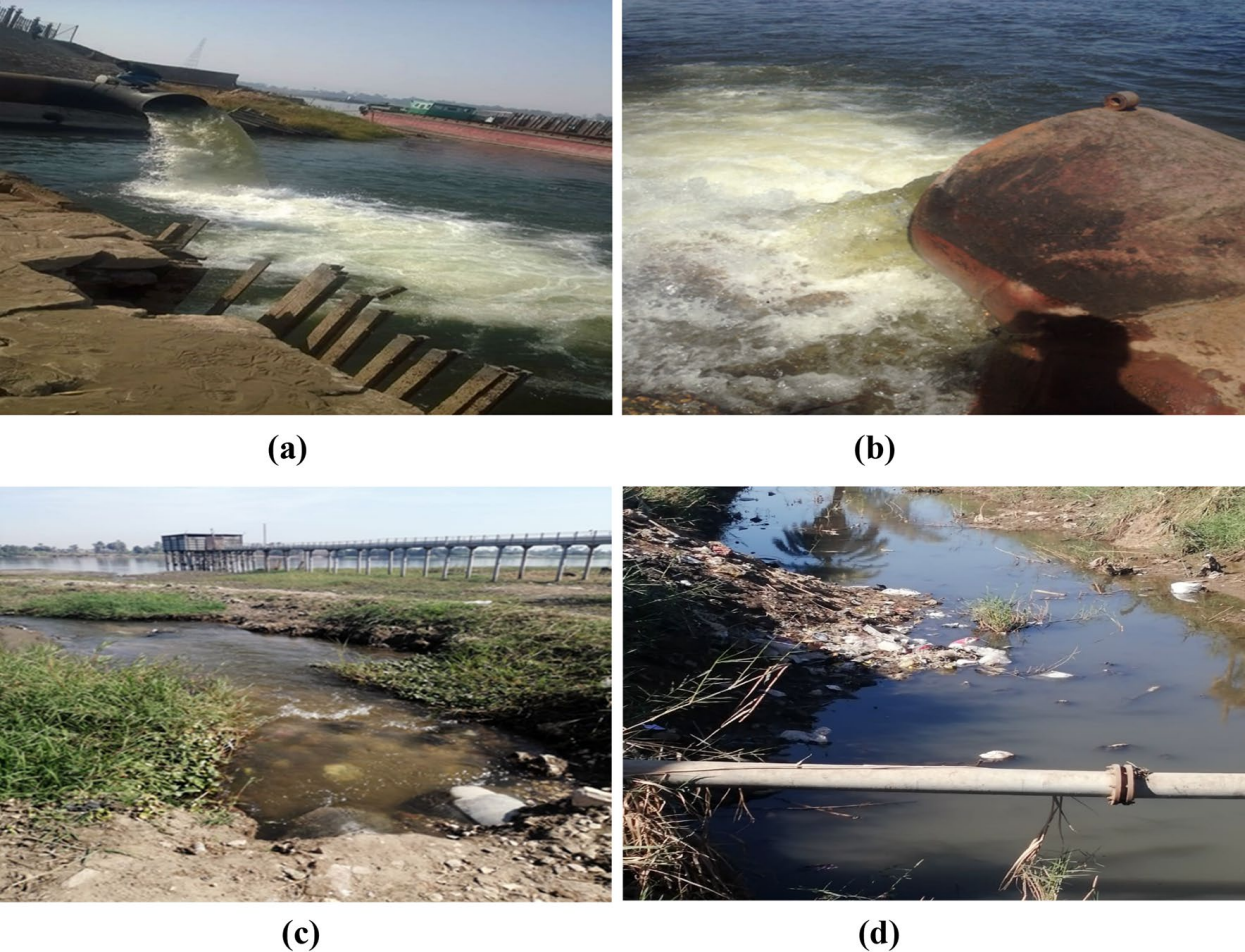
Wastewater disposal of into the Nile from sugar Cane factories at (**a**) Qus and (**b**) Nagi-Hamady, irrigation water disposal at (**c**) El-Mahrousa (**d**) El-Tramsa, drains in Qena governorate, south of Egypt (Camera photographs).

The activity concentrations of ^226^Ra, ^232^Th, and ^40^K in the Nile surface water (Bq L^−1^) and sediment (Bq kg^−1^) samples collected from Qena governorate are shown in Table 1. For water samples, ^226^Ra activities ranged from 0.08 ± 0.02 to 0.99 ± 0.09 Bq L^−1^, with an average value of 0.41 ± 0.05 Bq L^−1^. The concentrations ^232^Th and ^40^K varied from 0.05 ± 0.01 to 0.95 ± 0.09 and 2.52 ± 0.18 to 7.48 ± 0.49 Bq L^−1^, with averages 0.30 ± 0.04 and 4.73 ± 0.35 Bq L^−1^, respectively. The highest activity concentrations of ^226^Ra were found at Qus site (south Qena city; 0.98 Bq L^−1^) and Nagi-Hamady site (north Qena city; 0.99 Bq L^−1^). These values are more than twice the average value (0.41 Bq L^−1^). This may be attributed to the impact of industrial wastewater discharges from sugar factories at both sites into the Nile River (Fig. 2a,b). It’s estimated that the Nile receives 77,760 and 99,360 m^3^ day^−1^ wastewater from sugar factory drains at Qus and Nagi-Hamady, respectively ^39^.

**Table 1:** Activity concentration of natural radionuclides in Nile water and sediment along Qena governorate, south of Egypt.

Radionuclide	Nile water				Nile sediment			
	Minimum	Maximum	Average	Reference value^a^	Minimum	Maximum	Average	World average^b^
	Bq L^−1^				Bq kg^−1^			
^226^Ra	0.08 ± 0.02	0.99 ± 0.09	0.41 ± 0.05	1	9.30 ± 0.28	31.40 ± 1.38	18.14 ± 0.81	32
^232^Th	0.05 ± 0.01	0.95 ± 0.09	0.30 ± 0.04	1	6.70 ± 0.17	33.70 ± 1.96	17.98 ± 0.80	45
^40^K	2.52 ± 0.18	7.48 ± 0.49	4.73 ± 0.35		118.10 ± 10.8	695.10 ± 26.3	395.76 ± 19.30	420

^a^WHO reference values ^24^.

^b^UNSCEAR World average ^54^.

**Figure 3:**
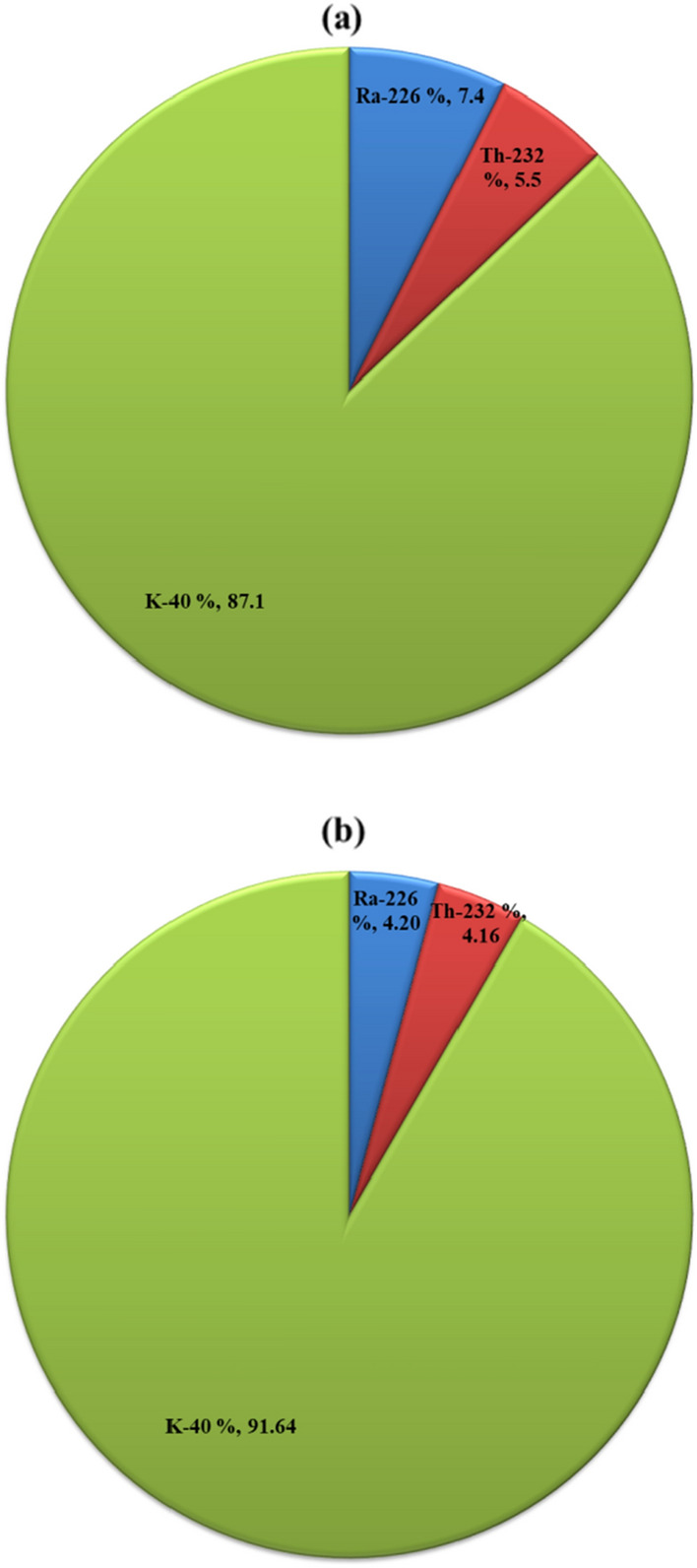
Percentage of natural radionuclides activitiies in (**a**) Nile water (**b**) Nile sediment, Qena, South of Egypt (prepared by Microsoft Excel software, 2010).

The maximum activity concentrations of ^232^Th and ^40^K were found in the surrounding Qena city sites, specifically: El-Mahrousa (^232^Th = 0.95 Bq L^−1^) and El-Tramsa (^40^K = 7.48 Bq L^−1^). These values are 3 times higher than the average of ^232^Th (0.3 Bq L^−1^) and 1.5 times higher than the average of ^40^K (4.73 Bq L^−1^). This may be attributed to the effects of irrigation water discharge at both sites (Fig. 2c,d), where inorganic potassium fertilizer used by farmers in nearby farmland can easily leach into the Nile through irrigation water, causing an increase in ^232^Th and ^40^K levels ^40^. Additionally, the geographic nature of Qena leads to a bend in the Nile stream channel, which increases erosion processes that may also increase ^232^Th and ^40^K levels ^41^. Figure 3a illustrated that ^40^K is the most abundant and contributes significantly to the total radionuclides activities in the Nile water compared to ^226^Ra and ^232^Th. The higher activity of ^226^Ra compared to that of ^232^Th activity in the studied water samples may be due to the high solubility of radium in water ^42^. Generally, the average activity concentrations follow the order ^40^K > ^226^Ra > ^232^Th.

Table 2 compares the results obtained with other studies from Egypt and the world. It was observed that the average activities of ^226^Ra, ^232^Th, and ^40^K in this study were higher than those observed in water samples from Assiut in Egypt ^43^ and Bakirçay River in Turkey ^6^ and lower than those from Al-Husseiniya River in Iraq ^44^. Also, a comparison with the WHO drinking water regulations indicated that the average values of ^226^Ra and ^232^Th do not exceed the WHO recommended limit of 1.0 Bq L^−1^ ^24^. Unfortunately, no limit value for ^40^K is included in the WHO report for comparsion.

**Table 2:** Natural radionuclides levels in water and sediment of the Nile River at Qena governorate, south of Egypt in comparison with similar studies.

Sample type	Location	Unit	^226^Ra	^232^Th	^40^K	References
Surface water	Nile River (Qena, Egypt)	Bq L^−1^	0.41	0.30	4.73	Present study
	Assiut, Egypt		0.20	0.08	0.69	^43^
	Nkalagu River, Nigeria		5.49	0.14	120.45	^40^
	Al-Husseiniya River (Karbala, Iraq)		1.90	1.23	10.08	^44^
	Bakirçay River, Turkey		0.18			^6^
	Euphrates river, Syria		0.48			^50^
Sediment	Nile River (Qena, Egypt)	Bq kg^−1^	18.14	17.98	395.76	Present study
	Nile River (Qena, Egypt)		14.44	15.02	197.58	^48^
	Nile River (Qena, Egypt)		29.34	42.69	117.90	^51^
	Nile River (EL- Mina, Egypt)		48.20	30.48	257.0	^52^
	Nile River (Rashid branch) Nile River (Damietta branch)		28.62 4.70	36.20 3.79	212.20 67.46	^49^
	Maritza, Turkey Tundja, Turkey		219 186	128 121	298 222	^1^
	Euphrates river, Syria		28.75		478.25	^50^
	Calabria, Italy		21.3	30.3	849.0	^13^
	Al-Husseiniya River (Karbala, Iraq)		15.8	11.2	311	^44^
	Rhumel River, Algeria		26.64	25.95	164.50	^53^

For sediment samples, ^226^Ra activities ranged from 9.30 ± 0.28 to 31.40 ± 1.38 Bq kg^−1^, with an average value of 18.70 ± 0.85 Bq kg^−1^. The concentrations of ^232^Th and ^40^K varied from 6.70 ± 0.17 to 33.70 ± 1.96 and 118.10 ± 10.80 to 695.10 ± 26.30 Bq kg^−1^, with averages of 18.40 ± 0.81 and 371.90 ± 20.10 Bq kg^−1^, respectively. The highest activity concentrations of ^226^Ra, ^232^Th, and ^40^K were found at El-Tramsa site with values of 31.40, 33.70, and 695.10 Bq kg^−1^, respectively. These values are 1.5 times higher than their corresponding averages. Also, high activities of ^226^Ra and ^232^Th were observed at Qus site, with values of 25.30 and 29.20 Bq kg^−1^, respectively. These findings suggested that the discharge of irrigation (El-Tramsa site) and industrial wastewater (Qus site) into the Nile raises the concentrations of the investigated radionuclides. Moreover, the results support the fact that sediments capture contaminants and act as ecological hosts for many pollutants entering water bodies ^45^. As shown in Fig. 3b, ^40^K, is more abundant in Nile sediment compared to ^226^Ra and ^232^Th, with the average activity following the order ^40^K > ^226^Ra > ^232^Th. The abundance of ^40^K is due to potassium being a major element in the earth's crust and in soil, where it is incorporated into mineral components ^46,47^. The high ^40^K activity may also be attributed to the sediment mineral composition, which includes feldspars, quartz, montmorillonite, illite, and kaolinite ^41^. A comparison of the results obtained with similar studies in Egypt and the world is presented in Table 2. The average activities of ^226^Ra, ^232^Th, and ^40^K were higher than those observed in previous studies for sediments from Qena ^48^ and Damietta ^49^, Egypt, and Al-Husseiniya River ^44^, Iraq, but lower than those observed for Euphrates River ^50^, Syria and Calabria River ^13^, Italy. Furthermore, the average activities of ^226^Ra and ^232^Th were lower than the reported values for Nile sediments from Qena ^51^, EL-Mina ^52^, and Rashid ^49^ in Egypt, and for sediments from Maritza, Tundja River^1^ in Turkey, and Rhumel River ^53^ in Algeria. In contrast, ^40^K exhibited higher activity compared to similar studies in Egypt and worldwide, except for those involving the Euphrates River, Syria, and Calabria River, Italy. On the other hand, the average values of ^226^Ra and ^232^Th align with the Egyptian soil averages 17 and 18 Bq kg^−1^, respectively, while ^40^K average is higher than the Egyptian average of 320 Bq kg^−1^. However, all average values of ^226^Ra, ^232^Th, and ^40^K are lower than the global average values of 32, 45, and 420 Bq kg^−1^, respectively ^54^.

### Heavy metals concentrations in Nile River

**Figure 4:**
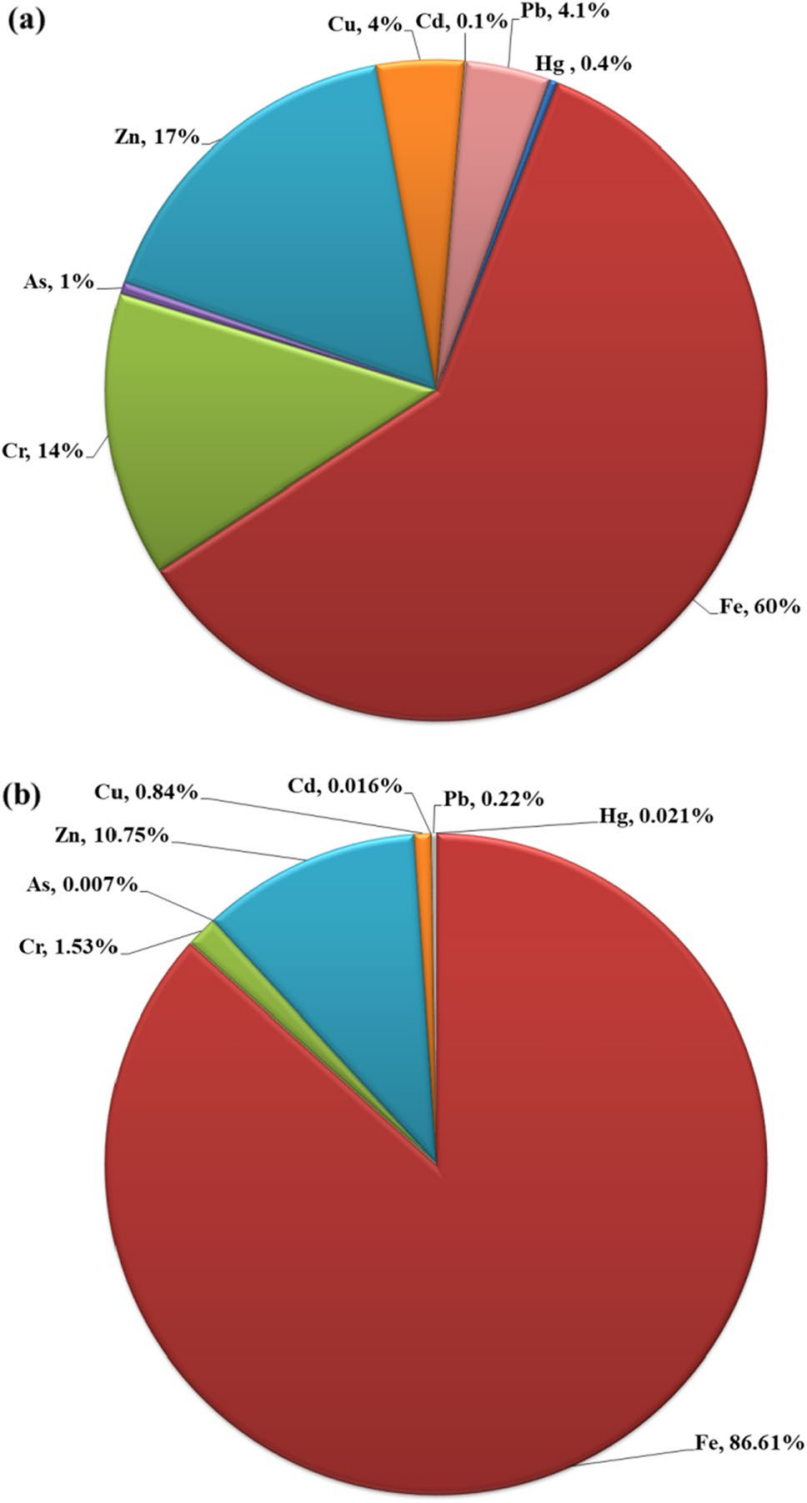
Percentage of Heavy metals concentrations in (**a**) Nile water (**b**) Nile sediment, Qena, South of Egypt (prepared by Microsoft Excel software, 2010).

Table 3 displays the concentrations of HMs (Hg, Fe, Cr, As, Zn, Cu, Cd, and Pb) in the analyzed samples. In the River water, the concentrations of Hg, Fe, Cr, Zn, Cu, Cd, and Pb range from 0.01 to 3.30, 0.10–566.0, 2.20–62.0, 0.04–9.27, 1.10–199.0, 0.50–98.90, 0.01–1.0, and 0.10–74.0 µg L^−1^, respectively. Significant variations in HMs concentrations were observed between sites, with detection rates (%) of 93, 60, 26, 26, 99, 84, 67, and 86 for Hg, Fe, Cr, Zn, Cu, Cd, and Pb, respectively. High concentrations of Hg, Fe, and Pb were detected at the Qus site (south Qena city), with values of 3.30, 566.0, and 74.0 µg L^−1^, respectively, which are 4 times greater than the averages for Hg and Fe and 8 times that of Pb. The El Shikhia site (south Qena city) exhibited high concentrations of Cr and Cd, with values of 62.0 and 1.0 µg L^−1^, which are 2 and 8 times greater than their respective averages. High concentrations of As, Zn, and Cu were observed at sites surrounding Qena city namely: Karm Omran (As: 9.27 µg L^−1^), Deir al-Sharqi (Zn: 199.0 µg L^−1^), and El Gramon (Cu: 98.2 µg L^−1^), these values are 7, 5, and 11 times greater than their respective averages. The high concentrations of HMs in these areas may be attributable to industrial and agricultural activities, as these activities generate untreated wastewater that is discharged into the Nile River. Based on these results, industrial and agricultural activities are among the main sources of heavy metals in the Nile River. As shown in Fig. 4a, the distribution of average HMs concentrations in the River water follows the order Fe > Zn > Cr > Cu > Pb > As > Hg > Cd.

**Table 3:** Heavy metals concentration in Nile water and sediment along Qena governorate, south of Egypt.

Heavy metal	Nile water				Nile sediment			
	Minimum	Maximum	Average	Drinking standard^a^	Minimum	Maximum	Average	Reference value^b^
	µg L^−1^				Mg kg^−1^			
Hg	0.01	3.30	0.81 ± 0.58	1.0	0.37	0.48	0.41 ± 0.04	0.06
Fe	0.10	566.0	121.0 ± 102.0	300.0	1340.0	2230.0	1670.0 ± 300.0	30,900
Cr	2.20	62.0	28.0 ± 17.80	50.0	20.0	51.0	29.40 ± 11.30	35.0
As	0.04	9.27	1.19 ± 0.91	10.0	0.11	0.20	0.14 ± 0.040	2.0
Zn	1.10	199.0	33.80 ± 26.10	1000.0	150.0	250.0	207.0 ± 33.0	52.0
Cu	0.50	98.20	8.62 ± 5.90	2000.0	2.75	52.70	16.20 ± 8.30	14.30
Cd	0.01	1.0	0.12 ± 0.07	3.0	0.03	0.75	0.31 ± 0.19	0.10
Pb	0.10	74.0	8.35 ± 6.70	10.0	1.18	9.92	4.32 ± 0.92	17.0

Average ± std.

^a^WHO drinking water standards ^24^.

^b^Reference values in Upper Continental Crust (UCC) ^27^.

In comparison to the drinking water quality standards ^24^, HMs concentrations in water samples from the studied sites mostly did not exceed the standard values, with only a few sites surpassing the standard values for Hg, Fe, Cr, and Pb. Previous studies of Nile River water from Qena and other areas in Egypt ^10,55,56^ have shown that our results are lower in terms of Fe, Cu, Cd, and Pb concentrations. Our findings are also consistent with research on surface water from various parts of the world ^5,40,57–59^ (Table 4).

**Table 4:** Heavy metals concentrations in water and sediment of the Nile River compared to similar studies.

Sample type	Location	Unit	Hg	Fe	Cr	As	Zn	Cu	Cd	Pb	References
Surface water	Nile River (Qena, Egypt)	µg L^−1^	0.813	121.0	28.0	1.19	33.80	8.62	0.12	8.35	Present study
	Nile River (Qena, Egypt)		NA	410.0	NA	NA	28.0	18.0	NA	24.0	^55^
	Nile River (Qena, Egypt)		NA	340.0	NA	NA	120.0	22.0	2.0	15.0	^56^
	Nile River (Qena, Egypt)		NA	1987.0	NA	NA	10.1	42.0	6.10	51.0	^10^
	Nile River (Assiut, Egypt)		NA	1440.0	NA	NA	30.0	25.0	5.40	21.0	
	Nile River (Cairo, Egypt)		NA	1180.0	NA	NA	55.0	45.0	NA	36.0	^55^
	Nile River (El-Menia, Egypt)		NA	620.0	NA	NA	75.0	35.0	NA	28.0	
	Terme River (Turkey)		NA	24.51	1.01	0.49	20.10	2.23	0.70	0.79	^5^
	Xiangjiang River, China		NA	18.0	0.30	5.55	16.80	NA	1.05	NA	^57^
	Nkalagu River, Nigeria		NA	NA	61.0	NA	NA	25.0	9.0	218.0	^40^
	Wen-Rui Tang River, China		0.03	NA	5.30	1.71	72.10	20.90	0.98	4.23	^58^
	Anikoko River, Ghana		154.0	NA	NA	325.0	NA	NA	230.0	0.20	^59^
Sediment	Nile River (Qena, Egypt)	Mg kg^−1^	0.41	1670.0	29.40	0.14	207.0	16.20	0.31	4.32	Present study
	Nile River (Aswan, Egypt) Nile River (Qena, Egypt) Nile River (Assiut, Egypt)		NA	400.0 380.0 470.0	NA	NA	101.0 91.0 100.0	0.03 0.02 0.03	0.40 0.50 0.60	3.10 3.10 4.40	^63^
	Nile River (Aswan, Egypt) Nile River (Esna, Egypt)		NA	22,090.0 37,920.0	NA	NA	52.0 73.0	15.70 32.20	NA	8.20 5.98	^60^
	Nile River (Egypt)		NA	11,350.0	3.90	NA	51.0	36.30	1.25	33.16	^61^
	Erfelek dam lake (Sinop, Turkey)		0.02	7342	39	1.26	14	6.1	0.1	4.6	^4^
	Anikoko River, Ghana		0.47	NA	NA	0.99	NA	NA	0.16	0.01	^59^
	Surma River, Bangladesh		NA	290.0	NA	NA	6.12	2.68	0.06	11.73	^62^
	Langat River, Malaysia		NA	NA	15.90	16.20	35.87	572.0	NA	30.44	^63^
	Xihe River, China		1.01	NA	35.60	11.20	205.0	45.30	2.88	21.88	^64^

*NA* not applicable.

In the River sediment, HMs concentrations (mg kg^−1^) ranged as follows: of Hg (0.37–0.48), Fe (1340.0–2230.0), Cr (20.0–51.0), As (0.11–0.20), Zn (150.0–250.0), Cu (2.75–52.70), Cd (0.03–0.75), and Pb (1.18–9.92). The Qus site displayed the highest concentrations of Hg, As, Cu, Zn, Cd, and Pb reflecting the influence of industrial activities on these elements in the River sediments. The highest concentrations of Fe and Cr were found in the outskirts of Qena city (Al-Waqf and Dandara), which could be attributed to the agricultural activities, the area’s geographical nature, and resulting erosion processes. The average HMs concentrations in Nile sediments followed the same order as in water, except for As (Fig. 4b). This suggests that the heavy metal sources in the study area may be primarily anthropogenic rather than natural. The average concentrations of the studied heavy metals in the Nile River sediments did not exceed their reference values in the upper continental crust provided by Wedepohl ^27^, with the exception of Hg, Zn, Cu, and Cd exceeding their reference values. The comparison with similar studies in Egypt and worldwide ^4,60–64^ showed that our results align with these studies (Table 4).

### Natural radionuclides and heavy metals correlations

Correlations between natural radionuclides and HMs were examined by calculating Pearson's correlation coefficients using Excel software. The Pearson correlation matrix (Table 5), revealed that values above 0.5 signify a significant correlation, whereas those below 0.5 indicate a non-significant correlation. Generally, varying correlation coefficients were observed among the studied elements, with more correlations found in sediment than in water. This may be due to the difference in the geochemical behavior of these elements in the environment. Significant positive correlation coefficients of Hg with both ^226^Ra and Pb in water and sediment (0.60 and 0.65; 0.60 and 0.56, respectively) suggest that these pollutants may have a similar source (anthropogenic activities) in the study area.

**Table 5:** Pearson correlation matrix of natural radionuclides and Heavy metals in water and sediments of Nile River, Qena, south of Egypt.

	^226^Ra	^232^Th	^40^K	Hg	Fe	Cr	As	Zn	Cu	Cd	Pb
Nile water											
^ 226^Ra	1										
^ 232^Th	0.32	1									
^ 40^K	**0.67**	0.46	1								
Hg	**0.60**	0.17	0.41	1							
Fe	− 0.10	− 0.05	− 0.07	0.02	1						
Cr	0.21	− 0.03	− 0.28	− 0.10	− **0.77**	1					
As	− 0.18	0.003	0.05	0.08	− 0.15	**0.51**	1				
Zn	− 0.03	0.03	0.01	− 0.22	0.03	− 0.24	− 0.32	1			
Cu	− 0.07	− 0.14	0.06	− 0.16	− 0.20	0.26	− 0.15	− 0.17	1		
Cd	− 0.02	− 0.02	− 0.03	0.19	0.19	0.30	− 0.15	− 0.16	− 0.04	1	
Pb	**0.63**	0.12	0.27	**0.60**	− 0.03	0.11	− 011	− 0.03	− 0.08	0.11	1
Nile sediment											
^ 226^Ra	1										
^ 232^Th	0.42	1									
^ 40^K	0.03	− 0.03	1								
Hg	**0.65**	0.36	**0.73**	1							
Fe	− **0.82**	− 0.36	0.31	− 0.19	1						
Cr	− 0.15	− 0.26	− 0.30	− **0.54**	− 0.40	1					
As	**0.95**	− 0.49	**0.77**	**0.86**	− 0.37	− 0.47	1				
Zn	− 0.26	0.38	**0.73**	0.37	0.34	− 0.08	0.15	1			
Cu	**0.72**	0.08	**0.61**	**0.93**	− 0.26	− 0.49	**0.99**	0.05	1		
Cd	− 0.06	**0.75**	0.46	0.38	0.15	− **0.68**	− 0.16	**0.89**	− 0.01	1	
Pb	0.08	**0.77**	**0.53**	**0.56**	0.16	− 0.47	− 0.05	**0.82**	0.21	**0.99**	1

Bold values refer to significant correlation, *p* < 0.05 (2-tailed).

### Heavy metal contamination of water samples

Water qualit**y** index (WQI) calculations were used to assess the suitability of the study samples as drinking water. WQI Values ranged from 0.04 to 259.40, with an average of 54.05. Among the 70 water samples, 44% were rated excellent to good, 40% were rated poor, and the remaining 16% were rated very poorly to unsuitable for drinking. The heavy metal evaluation index (HMEI) displayed an overall contamination trend in water, with values ranging from 0.02 to 10.87 and an average of 1.94. Based on HMEI thresholds^22,28^, 31% of the studied water samples were suitable for domestic usage, while 69% were unsuitable.

### Heavy metal contamination of sediment samples

**Figure 5:**
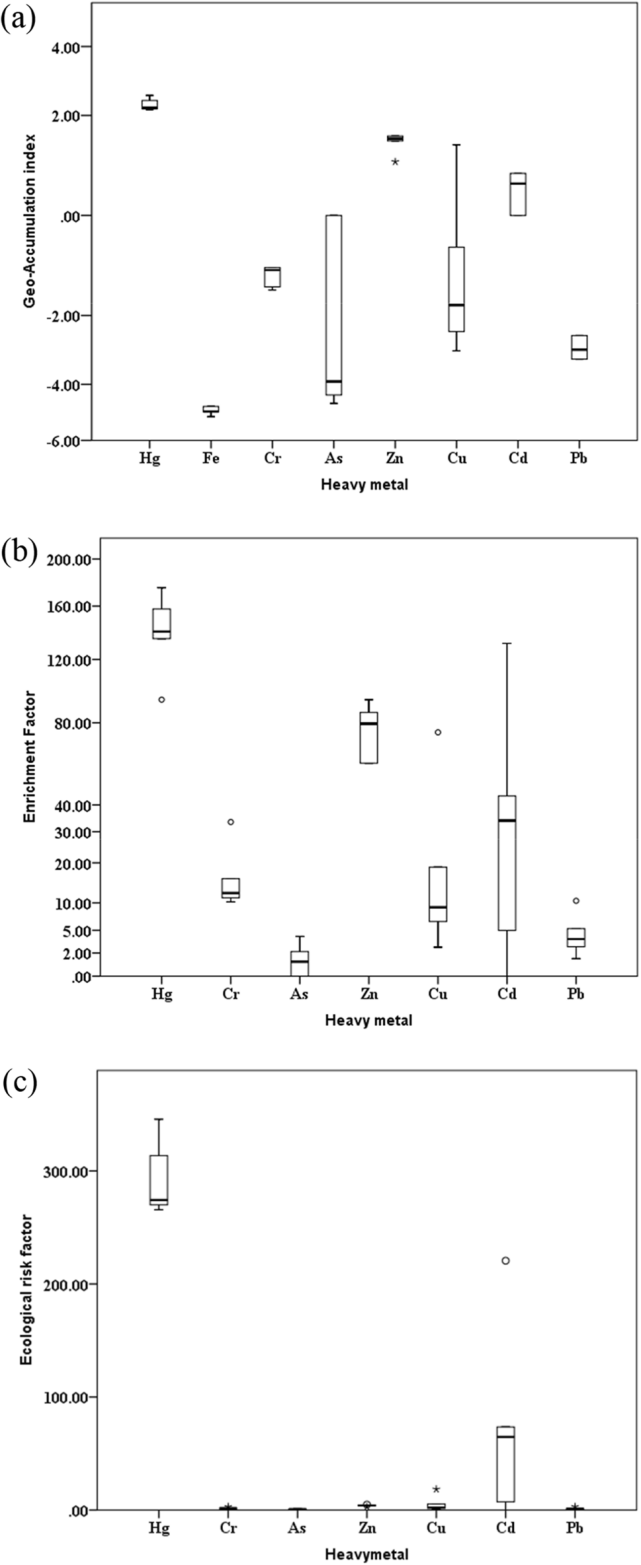
Contamination indices of sediment samples from Nile river at Qena governorate, south of Egypt (**a**) geo-accumulation index (**b**) Enrichment factor and (**c**) Ecological risk factor (prepared by IBM SPSS statistics 21).

Contamination indices for Nile sediments are shown in the box plot (Fig. 5). According to Geo-accumulation index values, Fe, Cr, As, Cu, and Pb levels were uncontaminated, while Zn and Cd exhibited moderate contamination, and Hg showed moderate to heavy contamination. Enrichment factor (EF) values revealed that all studied heavy metals had relatively low enrichment, with the exception of Hg and Zn, which were extremely severely enriched with EF values (58.5–93.70 for Zn; 93.71–175.07 for Hg) > 50 (Fig. 5b). The concentrations results of Hg and Zn reveal that they are relatively high in the Qus region, and gradually diminish toward Qena city. The reason may be attributed to the Nile receiving a direct discharge of industrial effluents from factories (sugar and fiber factories) in the Qus region which may be a source of Hg and Zn pollution. Of course, to find out the main reason for the increase of mercury and zinc concentrations in these sediments, further study is needed, such as studying the chemical and physical properties of the sediments, such as sediment texture, particle size, organic matter, pH, etc. Additionally, the ecological risk factor indicated that Hg was the only heavy metal posing a high ecological risk, with E_ri_ values < 320 (Fig. 5c).

### Radiological health risk

Table 6 summarizes the annual effective dose (AED) and excess lifetime cancer risk (ELCR) results for adults due to the ingestion of ^226^Ra, ^232^Th, and ^40^K from surface water and sediments of the Nile River. The results showed that the average AED due to the ingestion of ^226^Ra was higher than that of ^232^Th and ^40^K, which may be due to the higher radiotoxicity and solubility of ^226^Ra ^65,66^. The total AED for the studied Nile water (1.53 E − 01 ± 0.78 E − 01 mSv year^−1^) is slightly higher than the WHO reference limit (1.0 E − 01 mSv year^−1^) and significantly lower than the ICRP limit (1.0 E + 00 mSv year^−1^) for drinking water ^24,35^. Conversely, the total AED from sediment (4.26 E − 04 ± 1.43 E − 04) is much lower than the reference limits. The total ELCR from ingesting the studied radionuclides in water and sediments are 4.13 E − 04 ± 1.69 E − 04 and 2.96 E − 06 ± 1.04 E − 06, respectively, falling within the acceptable range of 1.0 E − 06 to 1.0 E − 04 for individual ELCR ^36^. These results indicate that the radiological hazard in the study area from the Nile River is minimal.

**Table 6:** Annual effective dose (AED) and excess lifetime cancer risk (ELCR) due to ingestion of natural radionuclides in Nile water and sediment from Qena governorate, south of Egypt.

Radionuclides	AED mSv year^−1^		ELCR	
	Water	Sediment	Water	Sediment
^226^Ra	8.23 E − 02 ± 5.06 E − 02	1.85E-04 ± 0.68 E − 04	2.11 E − 04 ± 1.30 E − 04	9.25 E − 07 ± 3.38 E − 07
^232^Th	4.97 E − 02 ± 4.15 E − 02	1.51 E − 04 ± 0.53 E − 04	4.12 E − 05 ± 3.44 E − 05	2.88 E − 07 ± 1.03 E − 07
^40^K	2.14 E − 02 ± 0.44 E − 02	8.96 E − 05 ± 3.73 E − 05	1.61 E − 04 ± 0.33 E − 04	1.75 E − 06 ± 0.67 E − 06
Total	1.53 E − 01 ± 0.78 E − 01	4.26 E − 04 ± 1.43 E − 04	4.13 E − 04 ± 1.69 E − 04	2.96 E − 06 ± 1.04 E − 06
Threshold limit	1.0 E − 01^a^		1.0 E − 06 to 1.0 E − 04^b^	

^a^WHO reference value ^24^.

^b^Acceptable range of ELCR ^36^.

### Health risk of metals

Table 7 presents the potential non-carcinogenic health risks for adults resulting from direct oral and dermal exposure to heavy metals in the Nile surface water and sediments from the Qena governorate, as estimated by chronic daily intake (CDI), hazard quotient (HQ), and hazard index (HI). The CDI results reveal that Zn and Fe are the most ingested metals, with average values of 5.69 E − 06 ± 1.03 E − 06 and 3.45 E − 06 ± 4.29 E − 06 for water, and 2.84 E − 07 ± 0.45 E − 07 and 2.29 E − 06 ± 0.42 E − 06 mg kg^−1^ day-1 for sediments, respectively. Conversely, Cd is the least ingested metal from both water and sediments, with average values of 3.45 E − 09 ± 5.77 E − 09 and 4.26 E − 10 ± 3.67 E − 10 mg kg^−1^ day^−1^, respectively. For dermal exposure to water and sediments, Fe displays the highest average values of 1.80 E − 08 ± 2.24 E − 08 and 2.88 E − 05 ± 0.52 E − 05 mg kg^−1^ day^−1^, respectively, while Cd exhibits the lowest averages of 1.80 E − 11 ± 3.01 E − 11 and 5.37 E − 09 ± 4.62 E − 09 mg kg^−1^ day^−1^, respectively. All the examined metals have HQ values below one, indicating that the CDI values are within their RfD. Additionally, the average HQ oral and HQ dermal values for all studied metals are below one, suggesting that exposure is within the acceptable non-carcinogenic health risk level. Furthermore, the average HI values are 1.52 E − 03 ± 5.44 E − 03 and 6.68 E − 06 ± 8.36 E − 06 for water ingestion, 1.89 E − 04 ± 2.67 E − 04 and 5.97 E − 02 ± 7.37 E − 02 for sediments ingestion, and dermal exposure, respectively. These values are also less than 1.0, indicating that the potential non-carcinogenic health risk from ingesting and dermally exposing to HMs in the Nile surface water and sediments at the Qena governorate is insignificant.

**Table 7:** Average chronic daily intake (CDI) mg kg^−1^ day^−1^, hazard quotients (HQ) and hazard index (HI) due to oral and dermal exposure of heavy metals of Nile water and sediment along Qena governorate, south of Egypt.

Heavy metal	CDI_oral_		CDI_dermal_		HQ_oral_		HQ_dermal_	
	Water sediment		Water sediment		Water sediment		Water sediment	
Hg	2.32 E − 08 ± 1.65 E − 08	5.64 E − 10 ± 0.59 E − 10	1.21 E − 10 ± 0.86 E − 10	7.10 E − 09 ± 0.75 E − 09	7.74 E − 05 ± 5.49 E − 05	1.88 E − 06 ± 0.19 E − 06	5.77 E − 06 ± 4.09 E − 06	3.38 E − 04 ± 0.36 E − 04
Fe	3.45 E − 06 ± 4.29 E − 06	2.29 E − 06 ± 0.42 E − 06	1.80 E − 08 ± 2.24 E − 08	2.88 E − 05 ± 0.52 E − 05	4.92 E − 06 ± 6.13 E − 06	3.26 E − 06 ± 0.59 E − 06	2.57 E − 04 ± 3.20 E − 04	4.11 E − 01 ± 0.75 E − 01
Cr	8.00 E − 07 ± 5.09 E − 07	4.03 E − 08 ± 1.54 E − 08	4.41 E − 09 ± 2.56 E − 09	5.07 E − 07 ± 1.94 E − 07	3.70 E − 03 ± 1.02 E − 03	1.34 E − 05 ± 0.51 E − 05	6.96 E − 05 ± 4.43 E − 05	8.46 E − 03 ± 3.24 E − 03
As	1.02 E − 06 ± 2.07 E − 06	5.89 E − 09 ± 1.66 E − 09	5.32 E − 09 ± 1.08E-08	6.01 E − 08 ± 3.43 E − 08	3.40 E − 03 ± 6.89 E -03	1.96 E − 05 ± 0.55 E − 05	4.33 E − 05 ± 8.77 E − 05	4.88 E − 04 ± 2.79 E − 04
Zn	5.69 E − 06 ± 1.03 E − 06	2.84 E − 07 ± 0.45 E − 07	3.02 E − 09 ± 3.23 E − 09	3.58 E − 06 ± 0.65 E − 06	3.22 E − 06 ± 3.44 E − 06	9.46 E − 07 ± 1.49 E − 07	5.04 E − 08 ± 5.38 E − 08	5.96 E − 05 ± 0.94 E − 05
Cu	2.46 E − 07 ± 4.55 E − 07	2.22 E − 08 ± 2.57 E − 08	1.29 E − 09 ± 2.38 E − 09	2.79 E − 07 ± 3.24 E − 07	6.16 E − 06 ± 1.14 E − 05	5.54 E − 07 ± 6.42 E − 07	1.07 E − 07 ± 1.98 E − 07	2.33 E − 05 ± 2.70 E − 05
Cd	3.45 E − 09 ± 5.77 E − 09	4.26 E − 10 ± 3.67 E − 10	1.80 E − 11 ± 3.01 E − 11	5.37 E − 09 ± 4.62 E − 09	3.45 E − 06 ± 5.77 E − 06	4.26 E − 07 ± 3.67 E − 07	1.80 E − 06 ± 3.01 E − 06	5.37 E − 04 ± 4.62 E − 04
Pb	2.38 E − 07 ± 3.90 E − 07	5.92 E − 09 ± 4.09 E − 09	1.24 E − 10 ± 2.04 E − 10	7.46 E − 08 ± 5.16 E − 08	6.81 E − 05 ± 1.12 E − 04	1.69 E − 06 ± 1.17 E − 06	5.53 E − 07 ± 9.05 E − 07	3.32 E − 04 ± 2.29 E − 04
HI					1.52 E − 03 ± 5.44 E − 03	6.68 E − 06 ± 8.36 E − 06	1.89 E − 04 ± 2.67 E − 04	5.97 E ± 7.37 E − 02

(average ± std).

Table 8 displays the average and total incremental lifetime cancer risk (ILCR) values for toxic metals Cr, As, Cd, and Pb due to oral and dermal contact with Nile surface water and sediments. The ILCR average values for Cr, As, Cd, and Pb were 4.00 E − 07 ± 2.55 E − 07, 1.53 E − 06 ± 3.10 E − 06, 1.31 E − 09 ± 2.19 E − 09, and 2.03 E − 09 ± 3.32 E − 09 from water ingestion, while values from sediment ingestion were 5.92 E − 09 ± 7.71 E − 09, 2.33 E − 09 ± 2.48 E − 09, 1.14 E − 10 ± 1.39 E − 10, and 2.65 E − 11 ± 3.48 E − 11, respectively. For dermal contact, the averages were 1.81 E − 07 ± 1.05 E − 07, 7.98 E − 09 ± 16.18 E − 09, 1.14 E − 10 ± 1.90 E − 10, and 5.23 E − 12 ± 8.55 E − 12 from water, and 6.11 E − 06 ± 7.96 E − 06, 4.35 E − 08 ± 5.15 E − 08, 2.39 E − 08 ± 2.91 E − 08, and 1.65 E − 08 ± 2.17 E − 08 from sediments, respectively. All mean and total ILCR values are below the cancer development risk threshold limit ^36^. As a result, the cancer development risk for adults from Cr, As, Cd, and Pb through oral and dermal exposure to Nile surface water and sediments in the study area is deemed negligible.

**Table 8:** Average incremental lifetime cancer risks (ILCR) of due to oral and dermal exposure of heavy metals of Nile water and sediment from Qena governorate, south of Egypt.

Heavy metal	ILCR_oral_		ILCR_dermal_	
	Water sediment		Water sediment	
Cr	4.00 E − 07 ± 2.55 E − 07	5.92 E − 09 ± 7.71 E − 09	1.81 E − 07 ± 1.05 E − 07	6.11 E − 06 ± 7.96 E − 06
As	1.53 E − 06 ± 3.10 E − 06	2.33 E − 09 ± 2.48 E − 09	7.98 E − 09 ± 16.18 E − 09	4.35 E − 08 ± 5.15 E − 08
Cd	1.31 E − 09 ± 2.19 E − 09	1.14 E − 10 ± 1.39 E − 10	1.14 E − 10 ± 1.90 E − 10	2.39 E − 08 ± 2.91 E − 08
Pb	2.03 E − 09 ± 3.32 E − 09	2.65 E − 11 ± 3.48 E − 11	5.23 E − 12 ± 8.55 E − 12	1.65 E − 08 ± 2.17 E − 08
ƩILCR	4.83 E − 07 ± 8.39 E − 07	8.39 E − 09 ± 7.96 E − 09	4.72 E − 08 ± 3.04 E − 08	6.20 E − 06 ± 2.55E-06

(average ± std).

## Conclusion

The Nile River's water quality is currently one of Egypt's most pressing issues, particularly due to increased industrial and agricultural activities and the impact of the Ethiopian dam on the river's water level. Consequently, this study aimed to estimate the levels of natural radionuclides (^226^Ra, ^232^Th, and ^40^K) and HMs (Hg, Fe, Cr, As, Zn, Cu, Cd, and Pb) in 70 surface water and 20 sediment samples from the Nile River along the Qena Governorate. Additionally, it sought to evaluate their environmental and health impacts using a sodium iodide detector and an atomic absorption spectrometer.

The average activity concentrations of ^226^Ra, ^232^Th, and ^40^K in water samples were 0.41 ± 0.05, 0.30 ± 0.04, and 4.73 ± 0.35 Bq L^−1^, while their activities in sediments were 18.70 ± 0.85, 18.40 ± 0.81, and 371.90 ± 20.10 Bq kg^−1^, respectively. Activity concentrations varied among sites, with higher values found at locations affected by industrial and/or agricultural activities. Generally, the average activity concentrations followed the order ^40^K > ^226^Ra > ^232^Th for both surface water and sediments. The average HMs concentrations in Nile water followed the order Fe > Zn > Cr > Cu > Pb > As > Hg > Cd, while their order in sediments was Fe > Zn > Cr > Cu > Pb > Hg > Cd > As, indicating Fe's dominance in the studied area. Hg had a significant correlation with both ^226^Ra and Pb, suggesting that these pollutants might have a common source (e.g., fertilizers) in the study area. Contamination indices of water and sediment samples revealed that 56% of water samples were unsuitable for drinking, and sediments were contaminated by Hg and Zn.

The annual effective dose for adults due to ingesting ^226^Ra, ^232^Th, and ^40^K from water was above the WHO permissible limit (0.1 mSv year^−1^) but below the ICRP limits (1.0 mSv year^−1^). The chronic daily intake of metals (CDI) showed that Zn and Fe were the most ingested metals, while Cd was the least. Values of HQ oral, HQ dermal, and HI for all studied metals were less than unity, indicating that exposure to the examined metals is within the acceptable level of non-carcinogenic health risk. Furthermore, ILCR values were below the threshold limit, indicating that the risk of developing cancer from Cr, As, Cd, and Pb through oral and dermal exposure is considered negligible. This study provides guidelines on natural radioactivity and HMs levels and their health effects on adults in the study area. Of course, the effect of industrial wastes and potassium fertilizers needs further study in future studies. So, continuous monitoring to obtain a comprehensive estimate of anthropogenic input to the Nile River is advised.

### Supplementary Information

Supplementary Tables.

## Data Availability

All data generated or analyzed during this study are included in this article and its supplementary file. The raw data supporting the conclusions of this article will be made available by the authors, without undue reservation.
