# Supplementary material for: Oral and dermal exposure to natural radionuclides and heavy metals in water and sediments of Nile River, Qena, southern Egypt
Source: Sci Rep. 2023 Dec 13;13:22098. doi: 10.1038/s41598-023-49389-3 (PMC10719346; doi:10.1038/s41598-023-49389-3)
Supplement: Supplementary file 1 — Supplementary Tables. [file 41598_2023_49389_MOESM1_ESM.docx]

| Metal | Certified value (mg/kg) | Measured value (mg/kg) | Recovery (%) |
| --- | --- | --- | --- |
|  |  |  |  |
| As | 30.30 | 27.27 | 90 |
| Cd | 2.23 | 2.04 | 91.5 |
| Cr | 90.60 | 83.35 | 92 |
| Fe | 41060 | 39417 | 96 |
| Cu | 326 | 303.12 | 93 |
| Pb | 188 | 180.30 | 95.9 |
| Hg | 2.98 | 2.65 | 89 |
| Zn | 376 | 349.04 | 92.8 |

**Table 1S.** Certified concentrations (mg/kg) of reference material (PACS-3, marine sediments), measured concentrations, and recoveries.

| *Igeo* value | Class^a^ | *EF* value | Class^b^ | *E*ri value | Class^c^ |
| --- | --- | --- | --- | --- | --- |
| *Igeo* < 0 | uncontaminated | *EF* < 2 | no enrichment | *E*ri < 40 | low potential ecological risk |
| 0 < *Igeo* < 1 | uncontaminated to  moderately contaminated | 2 < *EF* < 3 | minor enrichment | 40 ≤ *E*ri < 80 | moderate potential ecological risk |
| 1< *Igeo* < 2 | moderately contaminated | 3 ≤ *EF* ≤ 5 | moderate enrichment | 80 ≤ *E*ri < 160 | considerable potential ecological risk |
| 2 < *Igeo* < 3 | moderately to heavily contaminated | 5 ≤ *EF* ≤ 10 | moderate to severe enrichment | 160 ≤ *E*ri < 320 | high potential ecological risk |
| 3 < *Igeo* < 4 | heavily contaminated | 10 ≤ *EF* ≤ 25 | severe enrichment | *E*ri ≥ 320 | very high ecological risk |
| 4 < *Igeo* < 5 | heavily to extremely contaminated | 25 ≤ *EF* ≤ 50 | very severe enrichment |  |  |
| *Igeo* ˃5 | extremely contaminated | *EF* > 50 | extremely severe enrichment |  |  |

^a^ Muller ^[26]^, ^b^ Chen et al ^[31]^, ^c^ Khuzestani and Souri ^[30]^

**Table 2S.** Threshold values and corresponding contamination grades of I_geo_, EF**,** and Eri

| Parameter | Symbol | Unit | Ingestion | Dermal | Reference |
| --- | --- | --- | --- | --- | --- |
| ith Heavy metal concentration in water (or) sediment | C_i_ | mg L^-1^ (or)  mg kg^-1^ |  |  |  |
| Ingestion rate (water)  (sediment) | IR | L day^-1^ | 2 |  | [24] |
|  |  | mg day^-1^ | 100 |  | [33] |
| Exposure frequency | EF | day year^-1^ | 365 | 350 |  |
| Exposure duration | ED | year | 70 | 30 |  |
| Body weight for adult | BW | kg | 70 | 70 |  |
| Average time | AT | day | 25550 | 10500 | [22] |
| Exposed surface area (skin surface area) | SA | cm^2^ |  | 18000 | [33] |
| Permeability coefficient | K_p_ | cm h^-1^ |  | 0.001for Hg,Fe, Cr, As, Cu, Cd  0.0001 for Pb, 0.0006 for Zn |  |
| Conversion factor | CF | L cm^-3^ |  | 0.001 |  |
| Exposure time | ET | hour/event |  | 0.58 |  |
| Absorption factor | ABS |  | As=0.03, others= 0.001 | As=0.03, others= 0.001 |  |
| Reference dose of heavy metal due to ingestion or dermal exposure | RfD_in/d_ | mg kg^-1^ day^-1^ | Hg= AS= 0.0003, Fe= 0.7 , Cr =0.003, Zn =0.3, Cu =0.04, Cd= 0.001, Pb= 0.0035 | Hg = 0.000021,AS= 0.000012, Fe = 0.00007, Cr =0.00006, Zn =0.06, Cu =0.012, Cd= 0.00001, Pb= 0.00025 | [33, 38,65] |
| Cancer slope factor of heavy metals | CSF | (mg kg^-1^ day^-1^) | Cr=0.5, As=1.5, Cd=0.38, Pb=0.0085 | Cr=41, As= 1.5, Cd=6.3, Pb=0.043 |  |
| Cancer slope factor of radionuclides | CSF | pCi risk^-1^ | ^226^Ra=3.85E-10, ^232^Th= 1.01E-10, and ^40^K=2.47E-11 (for water ingestion)  ^226^Ra=7.29E-10, ^232^Th= 2.31E-10, and ^40^K=6.18E-11 (for sediment ingestion) |  | [33] |

**Table 3S.** Parameter used for human health risk calculations due to heavy metal and radionuclides exposure (ingestion and dermal) of Nile water and sediment
